# Lung function in young adults born small for gestational age at term

**DOI:** 10.1111/resp.14361

**Published:** 2022-09-29

**Authors:** Kilian Vellvé, Álvaro Sepúlveda‐Martínez, Mérida Rodríguez‐López, Francesca Crovetto, Gabriel Bernardino, Felip Burgos, Rosa Faner, Àlvar Agustí, Bart Bijnens, Eduard Gratacós, Fàtima Crispi, Isabel Blanco

**Affiliations:** ^1^ BCNatal—Barcelona Center for Maternal‐Fetal and Neonatal Medicine (Hospital Clínic and Hospital Sant Joan de Déu), Institut Clínic de Ginecologia, Obstetrícia i Neonatologia, Universitat de Barcelona, Centre for Biomedical Research on Rare Diseases (CIBER‐ER) Barcelona Spain; ^2^ Institut d'Investigacions Biomèdiques August Pi i Sunyer (IDIBAPS) Barcelona Spain; ^3^ Fetal Medicine Unit, Department of Obstetrics and Gynecology Hospital Clínico de la Universidad de Chile Santiago de Chile Chile; ^4^ Universidad Icesi Cali Colombia; ^5^ BCN Medtech, Department of Information and Communication Technologies Universitat Pompeu Fabra Barcelona Spain; ^6^ CREATIS, CNRS UMR5220, INSERM U1206, Université Lyon 1, INSA Lyon France; ^7^ Respiratory Institute, Hospital Clínic Universitat de Barcelona Barcelona Spain; ^8^ Centre for Biomedical Research on Respiratory Diseases (CIBERES) Barcelona Spain; ^9^ ICREA Barcelona Spain

**Keywords:** club cell protein, CO diffusion capacity, intrauterine growth restriction, lung capacity, prematurity, respiratory function tests, small for gestational age, surfactant protein

## Abstract

**See related** Editorial

To the Editors:

Moderate to extreme prematurity is associated with lower lung function in adults^1^ while evidence is poorer and controversial for late prematurity.^2^ Likewise, the potential long‐term impact on adult lung function of being born small for gestational age (SGA) at term is not well established since most previous studies in this field have been done in groups with participants enrolled by birthweight and not by SGA *per se*.^2^ This may be important because not all infants born SGA have experienced intrauterine growth restriction (IUGR) and, the other way round, early IUGR does not necessarily bring fetal growth down below the 10th percentile (the definition of SGA). We recently showed that young adults born SGA at term had markedly reduced exercise capacity, mostly of cardiovascular origin.^3^ In particular, they showed lower maximal workload, peak oxygen consumption and oxygen pulse, as well as higher minute ventilation/carbon dioxide production equivalent at the anaerobic threshold, than age‐matched controls.^3^ Here, we extend and complement these previously published observations^3^ with the analysis of pulmonary physiology (spirometry and carbon monoxide diffusing capacity [D_LCO_]) and the measurement of circulatory markers of abnormal lung development, including surfactant protein A and D (SP‐A and SP‐D) and club cell protein 16 (CC16).

We conducted an ambispective, controlled, cohort study whose detailed methodology has been published elsewhere.^3^ Briefly, from the birth records of our institution, we identified individuals *born at term* (≥37 weeks of gestation) between 1975 and 1995 with either appropriate weight for gestational age (AGA) or SGA (<10th centile for gestational age). Distinction between constitutionally small and growth‐restricted participants was not possible since Doppler ultrasound, which is the tool allowing to differentiate between these two groups, was not widely used when these individuals were born, so their medical records do not include this data. Exclusion criteria were twins, congenital malformations, genetic syndromes, macrosomia, mental disorder, current pregnancy or professional sports practice.

Demographics and medical history were recorded. Forced spirometry and D_LCO_ were determined following international standards. Reference values were those of Roca et al.^4^, ^5^ The serum concentrations of SP‐A, SP‐D and CC16 were determined using ELISA or Luminex following manufacturer's instructions.

Results are presented as mean ± SD, median [interquartile range—IQR] or number (%). Comparisons of perinatal and demographic data were performed using the Student's *t*‐test, Wilcoxon rank‐sum test, Chi‐square or Fisher's exact tests, as appropriate. Comparisons of pulmonary results were adjusted by age, sex, body surface area and smoking exposure using multivariate linear regression models. A *p*‐value < 0.05 was considered statistically significant. Analyses were performed using Stata/IC 15.1.

We compared individuals born at term with SGA (*n* = 61) or AGA (*n* = 66) in their early thirties (Table 1). All participants were of Caucasian origin and were born in Barcelona (Spain). By design, birthweight was lower in SGA. The number of males and females was similar in both groups. In adulthood, weight was similar but height was significantly lower in SGA. The proportion of smokers was also similar in both groups (Table 1). There were three AGA‐born and six SGA‐born individuals with asthma at the time of study (one and three requiring treatment, respectively) but this difference was not statistically significant. Although absolute values of forced expiratory volume in the first second (FEV_1_) were significantly lower in the SGA group, when expressed as % of reference values, spirometric and D_LCO_ values were similar and normal in both groups. Differences in absolute D_LCO_ values did not reach statistical significance but sample size was smaller. The circulating levels of SP‐A, SP‐D and CC‐16 were also similar in both groups. The results of the multivariate analysis did not show any further differences in the analysed parameters.

**TABLE 1:** Perinatal, demographic and pulmonary characteristics of the study population

	AGA (*n* = 66)	SGA (*n* = 61)	*p*‐value^a^
Perinatal data			
Birthweight (g)	**3388 [3210–3550]**	**2550 [2440–2700]**	***<0.001***
Gestational age at birth (weeks)	39.8 ± 1.1	40.1 ± 1.3	*0.115*
Birthweight centile	**53 [36–65]**	**2 [1–4]**	***<0.001***
Demographics			
Current age (years)	33.4 ± 3.6	34.4 ± 3.5	*0.151*
Female (%)	31 (47)	32 (53)	*0.536*
Height (m)	**1.72 ± 0.08**	**1.66 ± 0.09**	***<0.001***
Weight (kg)	74.5 [60–87]	67.5 [58–82]	*0.145*
Smoking habit (%)^b^	16 (24.2)	19 (31.2)	*0.384*
Pulmonary results at rest			
FEV_1_ (L)	**3.73 ± 0.66**	**3.40 ± 0.73**	***0.012***
FEV_1_ (% pred.)	95.9 ± 9.1	94.9 ± 10.6	*0.748*
FVC (L)	4.76 [4.06–5.37]	4.58 [3.42–5.19]	*0.193*
FVC (% pred.)	99.4 ± 9.6	100.3 ± 10.1	*0.565*
FEV_1_/FVC (%)	78.8 ± 6.1	77.8 ± 7.3	*0.432*
CO diffusing capacity of the lung^c^			
D_LCO_sb (ml/min/mm Hg)	28.7 ± 6.8	25.5 ± 6.2	*0.273*
D_LCO_ (% pred.)	88 ± 12	86 ± 12	*0.975*
VA (L)	5.64 [4.56–6.54]	5.72 [4.04–5.94]	*0.191*
VA (% pred.)	99 ± 10	100 ± 12	*0.648*
KCO (D_LCO_/VA)	5.05 ± 0.56	4.95 ± 0.71	*0.937*
KCO (% pred.)	92 ± 12	91 ± 16	*0.902*
Circulating biomarkers			
SP‐A (pg/ml)	704 [486–1270]	795 [523–1084]	*0.850*
SP‐D (pg/ml)	6.67 [4.64–10.8]	8.26 [3.50–11.9]	*0.630*
CC‐16 (ng/ml)	12.5 [9.60–19.1]	12.7 [9.75–15.9]	*0.509*

*Note*: Data shown as mean ± SD, median [IQR] or *n* (percentage). Bold is to highlight significant results. Italic for the statistical analysis.

^a^ *p*‐value adjusted for BSA, age, sex and current smoking status.

^b^ Smokers were those individuals who smoked on a regular basis (≥1–2 cig/day). Individuals who had stopped smoking at least 1 year before the study onset were labelled as non‐smokers.

^c^ Data available for 29 controls and 30 SGA.

The main and novel observations of this study show that young adults born SGA are significantly shorter than AGA (indicating some degree of tracking of SGA into adulthood), but their lung function (spirometry and D_LCO_) is normal once normalized for sex, age and height (Table 1). Collectively, these observations indicate that, at variances with prematurity,^1^ being born at term SGA is not associated per se with reduced lung function later in life. This would be in keeping with a previous analysis of this same cohort that recently showed that the ventilatory response to exercise in SGA subjects was normal^3^; interestingly, however, their cardiovascular response was not, featuring both decreased maximal workload and oxygen consumption.^3^ What mechanisms can explain this differential pulmonary and cardiovascular consequences of being born SGA at term are unclear and cannot be ascertained from our data, although cardiac limitation in SGA seems related to left ventricular sphericity and this may not apply to lung development.^3^ Further, it may be important to note in this context that SGA is an umbrella term that includes babies that are constitutionally small but had a normal fetal growth trajectory, and children who suffered from fetal insults that result in IUGR.^6^ In any case, our results indicate that future studies of adults born SGA at term should focus on those with a history of IUGR, as these are the only ones likely to have, potentially, impaired lung function.

On the other hand, previous reports in animal models of SGA have shown disturbed expression of surfactant proteins and vascular endothelial growth factor in the lung parenchyma^7^, ^8^ but, to our knowledge, no previous study has assessed circulating lung biomarkers in adults born SGA. In keeping with the normal physiology observed in young adults born SGA, we did not find significant differences between three pneumo‐proteins (SP‐A, SP‐D and CC16) previously described to be associated with reduced lung function.^9^ We cannot exclude, however, that lack of differences may also relate to the time elapsed from the prenatal insult or the kind of sample studied (lung tissue vs. circulating blood).

Among the strengths of this analysis is the availability of an adult cohort of AGA and SGA subjects born in the same centre to be analysed. Among its potential limitations, its relatively small size and the absence of a formal sample size calculation. Also, potential differences in other body composition measures (e.g., sitting height, lean muscle mass) is also a limitation that may need to be addressed in future studies. Finally, we acknowledge that our results correspond to a one observation in time and that trajectories over time would be of future interest too.

In conclusion, these observations show, for the first time to our knowledge, that being born SGA *at term* tracks into a shorter height in early adulthood but does not result in reduced lung function for that reduced height.

## AUTHOR CONTRIBUTION

**Bart Bijnens:** Formal analysis (equal); writing – review and editing (supporting). **Àlvar Agustí:** Conceptualization (equal); funding acquisition (equal); methodology (equal); supervision (equal); writing – original draft (equal); writing – review and editing (lead). **Rosa Faner:** Formal analysis (equal); funding acquisition (equal); methodology (equal); writing – original draft (supporting). **Isabel Blanco:** Conceptualization (equal); funding acquisition (equal); methodology (equal); supervision (lead); writing – original draft (equal); writing – review and editing (lead). **Fàtima Crispi:** Conceptualization (lead); funding acquisition (equal); methodology (equal); supervision (supporting); writing – original draft (equal); writing – review and editing (supporting). **Eduard Gratacós:** Conceptualization (equal); funding acquisition (equal); supervision (equal); writing – review and editing (supporting). **Mérida Rodríguez‐López:** Data curation (equal); writing – review and editing (supporting). **Álvaro Sepúlveda‐Martínez:** Data curation (equal); writing – review and editing (supporting). **Kilian Vellvé:** Data curation (equal); formal analysis (lead); writing – original draft (lead); writing – review and editing (equal). **Felip Burgos:** Data curation (equal); methodology (equal); writing – review and editing (supporting). **Gabriel Bernardino:** Formal analysis (equal); writing – review and editing (supporting). **Francesca Crovetto:** Supervision (supporting); writing – review and editing (supporting).

## CONFLICTS OF INTEREST

Gabriel Bernardino received the ITN Cardiofunxion grant (Grant agreement #642676) from the European Commission. Felip Burgos is a member of the Medical Graphics Corporation Diagnostics (MGCD) Scientific Advisory Board and received consulting fees.

## HUMAN ETHICS APPROVAL DECLARATION

The study was approved by the local Institutional Review Board (Hospital Clínic/IDIBAPS). Informed consent was obtained from all study participants upon enrolment.

## Data Availability

The data that support the findings of this study are available from the corresponding author upon reasonable request.

## References

[1] Bui DS , Perret JL , Walters EH , Lodge CJ , Bowatte G , Hamilton GS , et al. Association between very to moderate preterm births, lung function deficits, and COPD at age 53 years: analysis of a prospective cohort study. Lancet Respir Med. 2022. 10.1016/S2213-2600(21)00508-7 35189074

[2] Saad NJ , Patel J , Burney P , Minelli C . Birth weight and lung function in adulthood: a systematic review and meta‐analysis. Ann Am Thorac Soc. 2017;14:994–1004. 28362513 10.1513/AnnalsATS.201609-746SR

[3] Crispi F , Rodríguez‐López M , Bernardino G , Sepúlveda‐Martínez Á , Prat‐González S , Pajuelo C , et al. Exercise capacity in young adults born small for gestational age. JAMA Cardiol. 2021;6:1308–16. 34287644 10.1001/jamacardio.2021.2537 PMC8576583

[4] Roca J , Sanchis J , Agustí‐Vidal A , Segarra J , Navajas D , Rodriguez‐Roisín R , et al. Spirometric reference values for a mediterranean population. Bull Eur Physiopathol Respir. 1986;22:217–24. 3730638

[5] Roca J , Rodriguez‐Roisín R , Cobo E , Burgos F , Perez J , Clausen JL . Single‐breath carbon monoxide diffusing capacity (D_LCO_) prediction equations for a mediterranean population. Am Rev Respir Dis. 1990;141:1026–32. 2327636 10.1164/ajrccm/141.4_Pt_1.1026

[6] Arigliani M , Spinelli AM , Liguoro I , Cogo P . Nutrition and lung growth. Nutrients. 2018;10:10. 10.3390/nu10070919 PMC6073340 30021997

[7] Orgeig S , Crittenden TA , Marchant C , McMillen IC , Morrison JL . Intrauterine growth restriction delays surfactant protein maturation in the sheep fetus. Am J Physiol Lung Cell Mol Physiol. 2010;298:L575–83. 20097737 10.1152/ajplung.00226.2009

[8] Gortner L , Hilgendorff A , Bähner T , Ebsen M , Reiss I , Rudloff S . Hypoxia‐induced intrauterine growth retardation: effects on pulmonary development and surfactant protein transcription. Biol Neonate. 2005;88:129–35. 15908743 10.1159/000085895

[9] Guerra S , Halonen M , Vasquez MM , Spangenberg A , Stern DA , Morgan WJ , et al. Relation between circulating CC16 concentrations, lung function, and development of chronic obstructive pulmonary disease across the lifespan: a prospective study. Lancet Respir Med. 2015;3:613–20. 26159408 10.1016/S2213-2600(15)00196-4 PMC4640928

